# Precision Oncology in Non-Small Cell Lung Cancer: Integrating Molecular Diagnostics, Targeted Therapies, and Resistance Mechanisms

**DOI:** 10.3390/genes17091076

**Published:** 2026-09-07

**Authors:** Aleksandra Litkowska, Jan Wojtas, Kaja Nadulska, Grzegorz Kurec, Oliwia Burdan, Paweł Adam Krawczyk

**Affiliations:** 1 Student Scientific Association, Department of Pneumonology, Oncology and Allergology, Jaczewskiego 8 Street, 20-090 Lublin, Poland; litkowska.aleksandra@outlook.com (A.L.);; 2 The microGRAM Student Scientific Association, Department of Pharmaceutical Microbiology, Medical University of Lublin, 20-093 Lublin, Poland; 3 Immunology and Genetics Laboratory, Department of Internal Disease, Medical University of Lublin, 20-059 Lublin, Poland

**Keywords:** non-small cell lung cancer, precision oncology, molecular biomarkers, targeted therapy, next-generation sequencing, liquid biopsy, resistance mechanisms

## Abstract

Background: Precision oncology has significantly transformed the management of non-small cell lung cancer (NSCLC) through the integration of molecular diagnostics, targeted therapies, and biomarker-driven treatment selection. Advances in next-generation sequencing (NGS) and liquid biopsy have improved the identification of actionable molecular alterations and enabled dynamic monitoring of tumor evolution. Objective: To provide a structured narrative review of current evidence regarding actionable molecular biomarkers, diagnostic methodologies, targeted therapies, and future directions in NSCLC precision oncology, with a specific focus on conceptualizing acquired resistance mechanisms. Methods: A structured narrative literature review was conducted by searching PubMed and Google Scholar for English-language studies published between 2015 and 2026. Eligible publications included clinical trials, cohort studies, translational research, reviews, and clinical guidelines addressing molecular profiling, targeted treatments, diagnostic approaches, and resistance mechanisms in NSCLC. Results: A total of 99 foundational studies and clinical documents were analyzed. Key actionable biomarkers included *EGFR* (Epidermal Growth Factor Receptor), *ALK* (Anaplastic Lymphoma Kinase), *ROS1* (ROS Proto-Oncogene 1, Receptor Tyrosine Kinase), *KRAS* (Kirsten Rat Sarcoma Viral Oncogene Homolog), *RET* (Rearranged during Transfection), *MET* (Mesenchymal–Epithelial Transition Factor), *HER2* (Human Epidermal Growth Factor Receptor 2) and *NTRK* (Neurotrophic Tyrosine Receptor Kinase) alterations, along with emerging targets such as *NRG1* (Neuregulin 1) fusions. NGS emerged as the cornerstone of comprehensive molecular profiling, while liquid biopsy enabled longitudinal monitoring of tumor dynamics and resistance development. To organize the biological complexity of treatment failure, acquired resistance mechanisms were categorized into a three-tiered conceptual framework: target-centric genetic evolution (Tier 1), cellular plasticity and intratumoral heterogeneity (Tier 2), and non-genetic/microenvironmental adaptation (Tier 3). Targeted therapies significantly improved clinical outcomes compared with conventional chemotherapy; however, acquired resistance remained a major challenge across all tiers. Conclusions: Precision oncology in NSCLC is evolving from a biomarker-focused approach toward a dynamic framework integrating molecular diagnostics, targeted therapies, and continuous resistance monitoring. The proposed three-tiered resistance framework provides a structured basis for understanding treatment failure, guiding molecular reassessment at progression, and informing future adaptive therapeutic strategies to improve long-term patient outcomes.

## 1. Methodology

### 1.1. Study Design and Search Strategy

This structured narrative review was based on a structured literature search focusing on precision oncology in non-small cell lung cancer (NSCLC), including molecular biomarkers, targeted therapies, diagnostic strategies, and resistance mechanisms.

A structured literature search was performed in PubMed and Google Scholar for studies published between 2015 and 2026. Search terms included combinations of “NSCLC”, “precision medicine”, “molecular biomarkers”, “targeted therapy”, “EGFR”, “ALK”, “ROS1”, “KRAS G12C”, “RET”, “MET”, “HER2”, “NTRK”, “taletrectinib”, “zidesamtinib”, “zongertinib”, “sevebertinib”, “zenocutuzumab”, “liquid biopsy”, “ctDNA”, and “next-generation sequencing”. Additional searches addressed resistance mechanisms, including secondary mutations, bypass signalling pathways, tumor heterogeneity, cellular plasticity, and histologic transformation. Pivotal primary phase III trial publications and major clinical practice guidelines (e.g., NCCN, ESMO) were systematically prioritized.

### 1.2. Eligibility Criteria

Eligible studies included phase II/III clinical trials, cohort studies, translational research studies, systematic reviews, narrative reviews, and clinical guidelines published in peer-reviewed journals between 2015 and 2026. Only English-language articles with available full text or comprehensive clinical data were included.

Studies were excluded if they were not focused on NSCLC, lacked relevance to precision oncology, consisted solely of preclinical investigations without translational significance, were case reports, conference abstracts without full-text publications, duplicate publications, or non-English articles. Conference abstracts were generally excluded unless they presented pivotal, high-impact clinical trial outcomes or updated efficacy data not yet available in full-text journal articles.

### 1.3. Study Selection

Literature screening was conducted based on topic relevance, methodological quality, and clinical significance for precision oncology in NSCLC. Priority was given to pivotal phase II/III clinical trials, high-impact translational studies, and international clinical guidelines. After evaluating the retrieved literature, a total of 99 foundational studies, clinical trial reports, and consensus guidelines were selected and included in the narrative synthesis.

### 1.4. Data Extraction and Synthesis

Information regarding study design, molecular alterations, diagnostic methods, therapeutic strategies, resistance mechanisms, and clinical outcomes was extracted and synthesized narratively. Acquired resistance mechanisms were synthesized and categorized into a conceptual three-tiered framework (target-centric genetic evolution, cellular plasticity/heterogeneity, and non-genetic microenvironmental adaptation) to structure the biological complexity.

### 1.5. Limitations

This review is subject to several limitations, including restriction to English-language publications, potential publication bias, and reliance on full-text and trial data availability. Furthermore, heterogeneity among study designs and outcome measures prevented quantitative synthesis. As a structured narrative review, it does not include a formal risk-of-bias assessment or a registered protocol (e.g., PROSPERO).

## 2. Introduction

Lung cancer remains one of the leading causes of cancer-related mortality worldwide despite substantial advances in diagnosis and treatment [1,2]. Late-stage diagnosis and the molecular heterogeneity of the disease continue to limit therapeutic efficacy, particularly in advanced disease [1,2]. Although tobacco smoking remains the principal risk factor, the increasing incidence of lung adenocarcinoma in never-smokers further underscores the biological diversity of lung cancer [2].

Lung cancer is broadly classified into non-small cell lung cancer (NSCLC), which accounts for approximately 85% of cases, and small cell lung cancer (SCLC), representing the remaining 15% [2]. NSCLC comprises mainly adenocarcinoma, squamous cell carcinoma, and large cell carcinoma, whereas SCLC is characterized by rapid growth, high metastatic potential, and poor prognosis [2,3].

Before the implementation of molecular diagnostics, platinum-based chemotherapy was the standard treatment for advanced lung cancer, although its efficacy remained limited and was frequently associated with significant toxicity [2,4].

The introduction of molecular profiling and next-generation sequencing (NGS) has transformed the management of NSCLC by enabling the identification of actionable genomic alterations and the implementation of precision medicine [5]. Targeted therapies directed against epidermal growth factor receptor (EGFR), anaplastic lymphoma kinase (ALK), ROS proto-oncogene 1 (ROS1), rearranged during transfection (RET), mesenchymal–epithelial transition (MET), neurotrophic tyrosine kinase receptor (NTRK), Kirsten rat sarcoma virus oncogene homologue (KRAS), B-Raf proto-oncogene, serine/threonine kinase (BRAF), human epidermal growth factor receptor 2 (HER2), and emerging targets such as neuregulin 1 (NRG1) fusion proteins have substantially improved clinical outcomes in molecularly selected patients [5]. In parallel, immune checkpoint inhibitors targeting programmed cell death protein 1 (PD-1), programmed death-ligand 1 (PD-L1), and cytotoxic T lymphocyte antigen 4 (CTLA-4) have become an integral component of lung cancer treatment [1,5].

Consequently, molecular and immune biomarkers—ranging from oncogenic driver mutations to PD-L1 expression and tumor mutational burden (TMB)—are now central to therapeutic decision-making [1,5]. They enable precise patient stratification for both targeted agents and immunotherapies, while offering vital insights into treatment resistance mechanisms and dynamic disease evolution [1,5].

While current clinical guidelines provide actionable recommendations for initial biomarker testing and first-line treatment selection [6,7,8], clinical decision-making upon disease progression remains highly challenging. Existing reviews frequently present resistance as a simple binary list of “on-target” versus “off-target” mutations, offering limited prospective utility for clinical management [6,9].

To bridge this gap, this review provides a structured, synthesis-driven framework designed to translate complex resistance biology into actionable clinical decision-making. Specifically, we organize acquired resistance mechanisms into a novel, three-tiered conceptual model: Target-Centric Genetic Evolution (Tier 1), Cellular Plasticity and Intratumoral Heterogeneity (Tier 2), and Non-Genetic/Microenvironmental Adaptation (Tier 3). By integrating comprehensive genomic profiling, longitudinal liquid biopsy monitoring, and actionable diagnostic algorithms into this Tier 1–3 framework, this review aims to guide post-progression molecular reassessment and inform next-generation therapeutic strategies in NSCLC.

## 3. Molecular Characteristics of Non-Small Cell Lung Cancer

Driver mutations are key biological alterations that sustain tumor growth through constitutive activation of oncogenic signalling pathways. They include established actionable targets such as *EGFR* exon 19 deletions and p(Leu858Arg) substitution, *ALK* and *ROS1* rearrangements, as well as targetable alterations such as p.(Gly12Cys) substitution in the *KRAS* gene. Other genetic abnormalities, including rare mutations in *EGFR* and *KRAS*, alterations in *BRAF* and *MET*, and rearrangements involving *NTRK* and *RET*, kinase domain alterations in HER2, as well as oncogenic fusions involving *NRG1*, may also qualify NSCLC patients for molecularly targeted therapies [6,10]. Importantly, these driver alterations are dynamic and may evolve under therapeutic pressure, contributing to tumor escape and disease progression [6].

**Figure 1:**
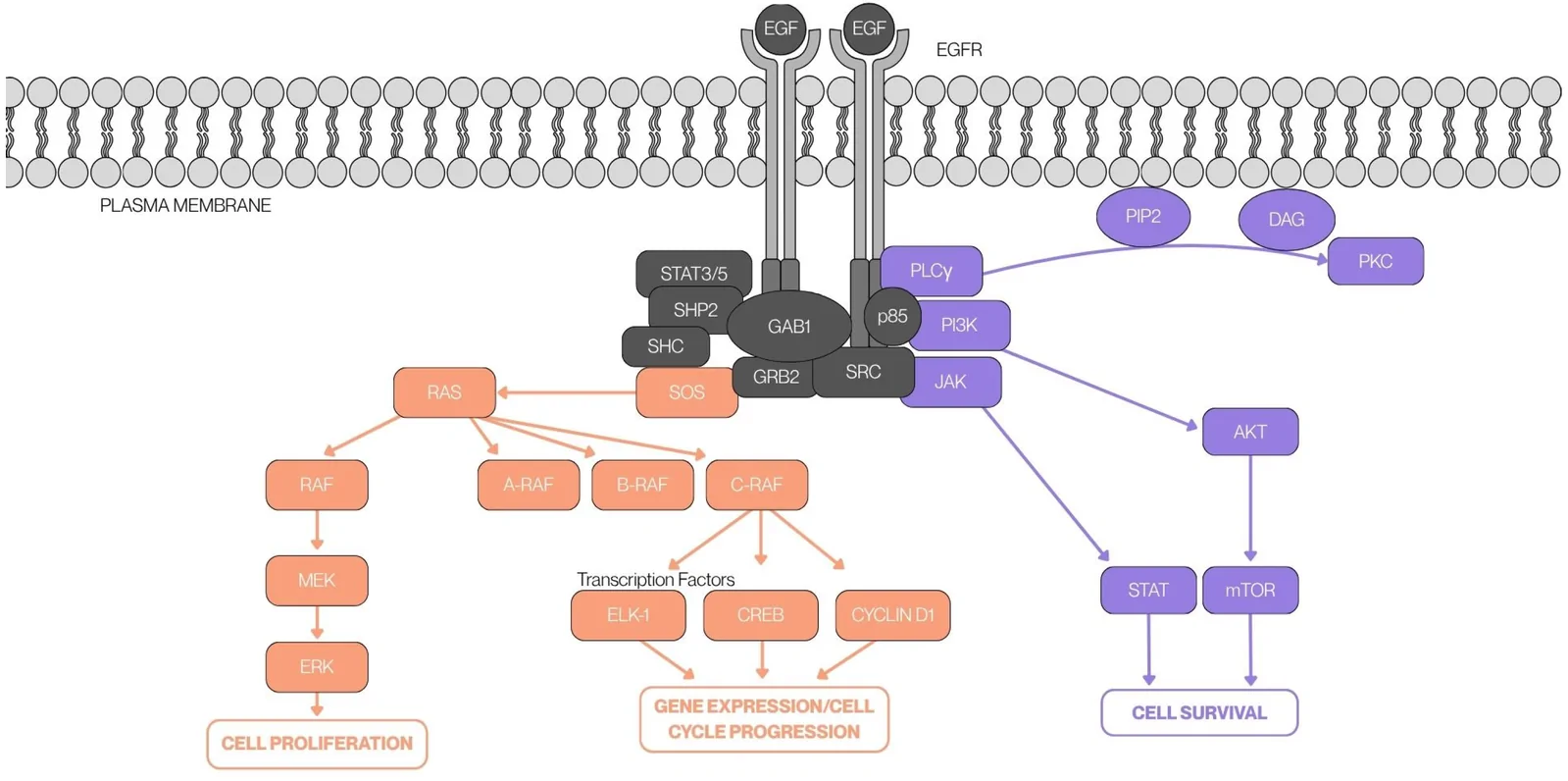
Representative receptor tyrosine kinase (RTK) downstream signalling cascades in NSCLC.

Oncogenic signalling in NSCLC is primarily mediated through interconnected cascades, particularly the RAS/MAPK (rat sarcoma virus/mitogen-activated protein kinase) and PI3K/AKT (phosphoinositide 3-kinase/protein kinase B) pathways. The RAS/MAPK pathway promotes proliferation through sequential activation of RAS, RAF (rapidly accelerated fibrosarcoma), MEK (mitogen-activated protein kinase kinase), and ERK (extracellular signal-regulated kinase), whereas the PI3K/AKT pathway regulates cell survival and metabolic adaptation. Furthermore, ERBB family receptor dimerization—such as HER2 activation or NRG1-mediated HER2/HER3 heterodimerization—similarly engages these downstream survival axes [10]. The interaction between these pathways creates a compensatory signalling network in which inhibition of one axis may activate alternative mechanisms, contributing to therapeutic resistance and the emergence of drug-tolerant persister (DTP) cells (Figure 1) [7].

Tumor heterogeneity represents a major challenge in NSCLC, as genetically distinct subclones may coexist within primary tumors and metastatic lesions. This diversity contributes to variable treatment responses and facilitates the emergence of resistant populations during therapy [11]. Importantly, the presence of a single driver mutation may not fully predict therapeutic response. In *KRAS*-mutant NSCLC, co-occurring alterations in *TP53* (tumor protein 53), *STK11* (serine/threonine kinase 11), and *KEAP1* (kelch-like ECH-associated protein 1) define biologically distinct molecular subgroups with different tumor behaviours and treatment sensitivities [12].

Given limited tissue availability in advanced disease, next-generation sequencing (NGS) is increasingly incorporated into molecular diagnostics. NGS enables simultaneous detection of driver mutations and co-alterations, improving molecular characterization and supporting treatment selection in patients with advanced NSCLC [12].

EGFR activation serves as a prototypical RTK model illustrating key downstream cascades, including the RAS/RAF/MEK/ERK, PI3K/AKT/mTOR, and PLCγ (phospholipase C gamma) pathways. Similar downstream networks are engaged by other oncogenic drivers in NSCLC, including HER2, ALK, ROS1, RET, MET, and NRG1 fusion proteins, driving cell proliferation, survival, and adaptive resistance.

## 4. Molecular Biomarkers and Precision Oncology in NSCLC

Targeted therapy represents a central component of modern precision oncology, focusing on molecularly defined oncogenic drivers responsible for tumor initiation and progression in NSCLC [10,13,14]. Therefore, comprehensive molecular profiling to identify actionable genomic alterations has become an essential prerequisite for treatment selection. Clinically relevant alterations include abnormalities in *EGFR*, *ALK*, *ROS1*, *BRAF*, *KRAS*, *HER2*, *MET*, *RET*, *NRG1*, and *NTRK* genes, allowing molecularly selected patients to receive targeted therapies [9,10,15]. Unlike conventional chemotherapy, targeted therapies selectively inhibit tumor-specific signalling dependencies, improving treatment precision and reducing systemic toxicity [13,14].

**Figure 2:**
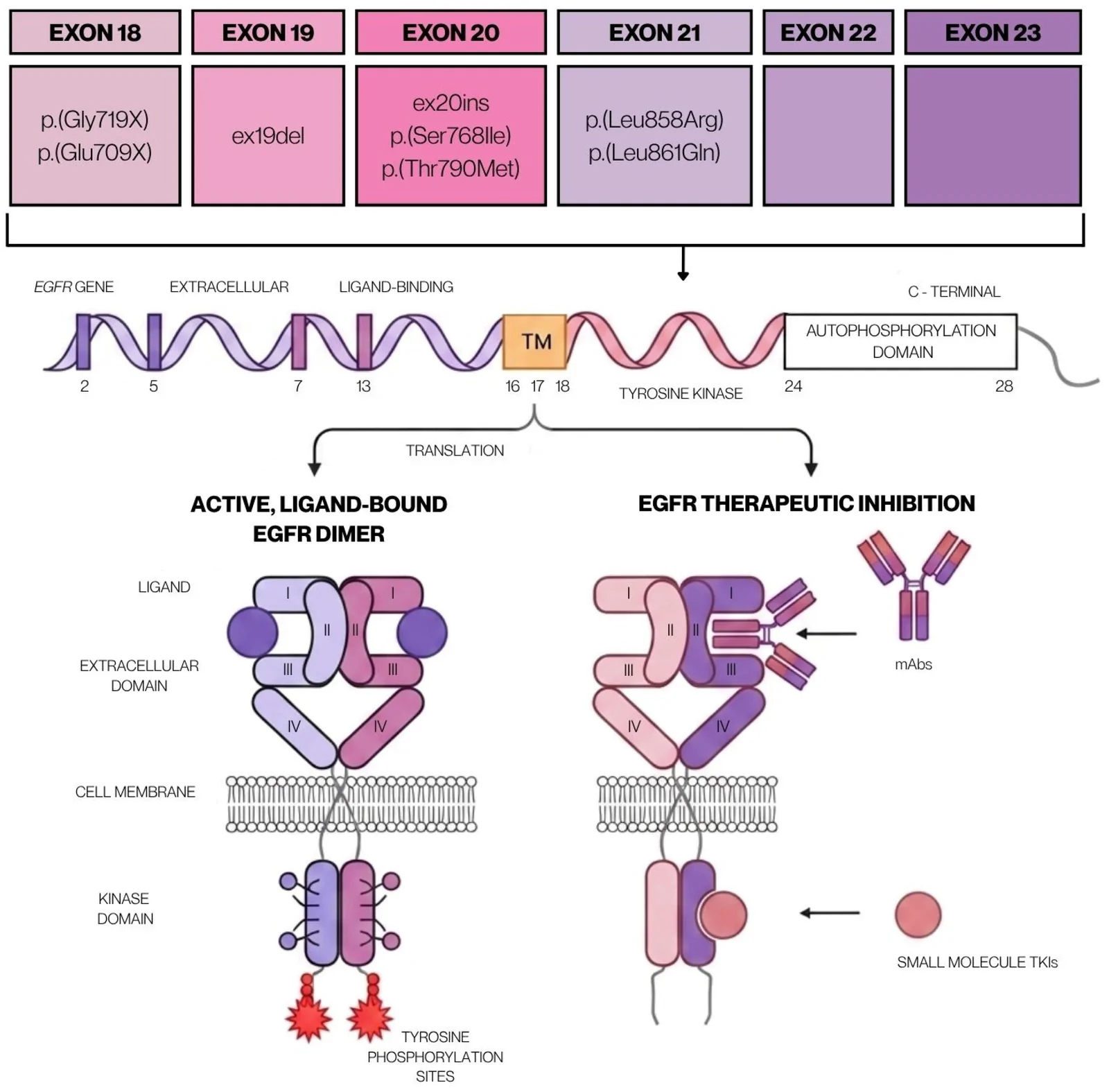
Structural overview of the *EGFR* gene and EGFR protein domain organization during therapeutic inhibition. Clinically relevant *EGFR* regions include exons 18–21 associated with therapeutic response and acquired resistance. Small-molecule TKIs selectively target the intracellular tyrosine kinase domain, whereas monoclonal and bispecific antibodies (e.g., amivantamab) target extracellular domains and induce receptor degradation.

Within this therapeutic framework, tyrosine kinase inhibitors and serine-threonine kinase inhibitors represent the most established targeted treatment strategies [8,16]. Tyrosine kinases regulate key processes involved in tumor development, including proliferation, survival, angiogenesis, and metastatic progression [10,16]. In NSCLC, oncogenic activation of receptor tyrosine kinases leads to persistent signalling through interconnected pathways, particularly RAS/MAPK and PI3K/AKT, which regulate tumor growth and survival [17,18]. EGFR tyrosine kinase inhibitors (EGFR-TKIs) inhibit receptor kinase activity, suppressing downstream signalling required for tumor maintenance and inducing growth inhibition in sensitive tumors (Figure 2) [16,18,19].

EGFR is a transmembrane receptor tyrosine kinase belonging to the *ErbB* (Erythroblastic leukemia viral oncogene homolog B) family that regulates cellular proliferation, survival, and metastatic progression. Activating *EGFR* mutations induce constitutive signalling through PI3K/AKT and RAS/MAPK pathways, contributing to oncogenic transformation. *EGFR*-mutant NSCLC represents a distinct molecular subtype, most commonly associated with adenocarcinoma and enriched among females, never-smokers, and Asian populations. Exon 19 deletions and exon 21 p.(Leu858Arg) substitution account for approximately 85–90% of *EGFR* mutations, with frequencies varying between populations, occurring in approximately 10–15% of Caucasian patients and up to 40–50% of Asian populations [2,17,20].

*EGFR* mutation testing is performed using PCR (polymerase chain reaction)-based assays and NGS, while *EGFR* alterations may also be detected in circulating tumor DNA (ctDNA) using liquid biopsy approaches. Current guidelines recommend molecular testing in all patients with advanced non-squamous NSCLC, with NGS increasingly preferred because it enables simultaneous detection of multiple genomic alterations [21,22].

EGFR-TKIs have significantly improved outcomes in patients with *EGFR*-mutant NSCLC. First-generation agents (gefitinib, erlotinib) and second-generation agents (afatinib, dacomitinib) improved progression-free survival (PFS) compared with chemotherapy. The third-generation inhibitor osimertinib further improved progression-free and overall survival while demonstrating superior central nervous system (CNS) activity, establishing it as the first-line standard of care following the FLAURA trial [17,23]. Subsequent clinical trials further expanded the role with osimertinib combined with chemotherapy compared with osimertinib monotherapy [24]. Furthermore, the MARIPOSA trial showed that a regimen containing amivantamab (a bispecific anti-EGFR and anti-MET antibody) and lazertinib (a third-generation EGFR-TKI) was more effective than osimertinib monotherapy in reducing the risk of disease progression and death [24,25]. Amivantamab combined with chemotherapy is also approved for patients with *EGFR* exon 20 insertions and in those with acquired resistance to osimertinib [24,26]. In addition, osimertinib has demonstrated clinical benefit in the adjuvant setting after surgical resection of early-stage NSCLC (ADAURA trial) and as consolidation therapy after chemoradiotherapy in locally advanced NSCLC (LAURA trial) [27,28].

Despite substantial initial efficacy, resistance to EGFR-TKIs inevitably develops. Following first- and second-generation EGFR-TKIs, the most common resistance mechanism is the emergence of the p.(Thr790Met) mutation in exon 20 of *EGFR*, occurring in approximately 50–60% of cases. Although osimertinib effectively overcomes p.(Thr790Met)-mediated resistance, secondary mechanisms frequently emerge, including the p.(Cys797Ser) tertiary mutation in *EGFR*, *MET* amplification, *HER2* alterations, and histological transformation to small cell lung cancer, reflecting dynamic adaptive evolution [29,30].

*ALK* rearrangements occur in approximately 3–7% of NSCLC cases, most commonly as *EML4* (echinoderm microtubule-associated protein-like 4)::*ALK* fusions. These alterations result in constitutive kinase activation and downstream signalling, defining a distinct molecular subtype frequently observed in younger patients, individuals with adenocarcinoma, and never-smokers [31]. *ALK* testing includes fluorescence in situ hybridization (FISH), immunohistochemistry (IHC), and NGS approaches. While FISH was historically considered the standard method, IHC is widely used for screening, whereas NGS enables comprehensive identification of fusion variants and additional genomic co-alterations [32]. ALK-positive NSCLC demonstrates high sensitivity to sequential ALK TKI therapy. Crizotinib was the first effective targeted agent, followed by second-generation inhibitors such as alectinib, brigatinib, ceritinib, and ensartinib, which substantially improved systemic and CNS efficacy. Lorlatinib, a macrocyclic third-generation inhibitor, provides robust activity against a broad spectrum of secondary *ALK* resistance mutations and superior CNS penetration. Based on the long-term results of the CROWN trial, lorlatinib is currently considered a preferred first-line treatment option for ALK-positive NSCLC [33]. Resistance mechanisms include secondary *ALK* kinase domain mutations (e.g., G1202R), *ALK* gene amplification, and bypass pathway activation involving *MET* or *EGFR*.

*ROS1* rearrangements occur in approximately 1–2% of NSCLC cases and define another actionable molecular subtype with clinical features similar to ALK-positive disease [34]. *ROS1* fusions are routinely detected using NGS or FISH [32,34]. Crizotinib and entrectinib demonstrate substantial initial clinical activity in ROS1-positive NSCLC, while repotrectinib has expanded therapeutic options by providing superior activity against CNS metastases and overcoming selected solvent-front resistance mutations (e.g., G2032R) [35,36,37]. Furthermore, novel next-generation ROS1 inhibitors, including taletrectinib and zidesamtinib (NVL-520), have demonstrated impressive intracranial responses and high potency against refractory *ROS1* resistance mutations, offering valuable therapeutic options upon progression [35,36,38]. Selective RET inhibitors (selpercatinib and pralsetinib) and TRK inhibitors (larotrectinib and entrectinib) have established high clinical response rates and durable disease control in patients harboring *RET* fusions and *NTRK1/2/3* gene fusions, respectively [9,10,15].

*KRAS* mutations occur in approximately 30–40% of lung adenocarcinomas. The p.(Gly12Cys) substitution (*KRAS* G12C) constitutes approximately 40% of all *KRAS* mutations (~13% of all lung adenocarcinomas) and shows a strong association with smoking history [39]. The clinical development of covalent allele-specific KRAS G12C inhibitors, including sotorasib and adagrasib, has successfully targeted this historically “undruggable” driver [40,41,42,43]. To address resistance driven by bypass signaling or non-G12C mutations, pan-KRAS inhibitors and RAS-ON selective agents are currently undergoing clinical evaluation [44,45,46].

In *BRAF*-mutant NSCLC, patients harboring the p.(Val600Glu) substitution benefit from dual BRAF/MEK inhibition (dabrafenib plus trametinib, or encorafenib plus binimetinib), whereas selective MET inhibitors (tepotinib and capmatinib) demonstrate significant efficacy in tumors with *MET* exon 14 skipping alterations [10,15,47,48,49].

The therapeutic paradigm for *HER2* (ERBB2) alterations has rapidly evolved. Antibody–drug conjugates (ADCs), specifically trastuzumab deruxtecan (T-DXd), have established a benchmark standard for *HER2*-mutated advanced NSCLC [48,49]. Additionally, novel selective HER2 TKIs—such as zongertinib and sevebertinib—have emerged as highly potent oral agents that selectively target *HER2* exon 20 insertions and kinase domain mutations while sparing wild-type EGFR, significantly reducing EGFR-related toxicities [10,15,50,51].

For oncogenic *NRG1* gene fusions, which drive HER2/HER3 heterodimerization and persistent survival signaling, the bispecific antibody zenocutuzumab has demonstrated meaningful clinical response rates, representing a novel target-specific therapy in driver-negative or rare fusion-driven NSCLC [9,10,52].

Compared with conventional platinum-based chemotherapy, targeted therapies selectively inhibit oncogenic signalling pathways, resulting in favorable overall tolerability and enabling prolonged treatment exposure [8,13,14]. Nevertheless, targeted agents remain associated with distinct class-specific adverse events. Skin rash, diarrhea, paronychia, mucositis, and fatigue are common during EGFR inhibition, whereas hepatotoxicity, cardiotoxicity, QTc prolongation, and interstitial lung disease (ILD) represent less frequent but potentially life-threatening complications requiring proactive monitoring and dose adjustments [9,47,53,54].

Despite substantial initial responses, acquired drug resistance remains the principal limitation to durable clinical benefit [16,18,19]. Adaptive resistance across molecular subtypes commonly involves secondary target mutations, bypass signaling pathway reactivation, cellular plasticity, and tumor heterogeneity.

Liquid biopsy analyzing circulating tumor DNA (ctDNA) has established a pivotal role in longitudinal molecular monitoring [55]. While tissue-based re-biopsy remains essential for diagnosing histologic transformation (e.g., SCLC transformation), ctDNA analysis offers a non-invasive tool to capture spatial and temporal intratumoral heterogeneity, enabling early detection of emerging resistance mechanisms (Tier 1–3) prior to radiologic disease progression [55,56,57,58,59].

## 5. Molecular Biomarker Diagnostics

Modern precision oncology integrates clinical, imaging, pathological, and molecular data, with comprehensive genomic profiling representing a fundamental component of therapeutic decision-making [60]. In non-small cell lung cancer, qualification for targeted therapies depends on accurate identification of actionable driver alterations and co-occurring genomic events [60]. Routine molecular biomarker testing has therefore transformed clinical practice by improving patient stratification and prediction of therapeutic response [61].

Despite these advances, most NSCLC cases are still diagnosed at advanced stages, highlighting the need for diagnostic approaches that enable sensitive and dynamic assessment of tumor biology throughout disease evolution [62].

Tissue biopsy, including core needle biopsy, fine-needle biopsy, and surgical specimens, remains the diagnostic gold standard for initial diagnosis and histologic classification [63,64]. However, its clinical utility is often limited by spatial sampling bias, an inability to fully capture intratumoral heterogeneity, procedure-related risks, and restricted feasibility for repeated longitudinal assessment, particularly in patients withanatomically inaccessible lesions or poor performance status [61,63,64].

Liquid biopsy (LB) has emerged as a complementary minimally invasive approach based on the analysis of tumor-derived material circulating in body fluids, most commonly peripheral blood [61,63,64]. It enables the assessment of circulating tumor cells (CTCs), ctDNA, microRNAs (miRNAs), circular RNAs (circRNAs), exosomes, nucleosomes, and epigenetic signatures, providing a broader representation of tumor heterogeneity than a single tissue specimen [61,63]. Clinically, liquid biopsy is particularly valuable for longitudinal monitoring, including detection of minimal residual disease (MRD) and emerging resistance alterations during targeted therapy, such as *EGFR* and *ALK* resistance mechanisms [63,64]. However, its sensitivity remains limited in early-stage disease, in tumors with low ctDNA shedding, and in routine practice where access to advanced platforms varies.

The selection of appropriate analytical diagnostic methodologies is crucial for accurate molecular characterization. Quantitative real-time polymerase chain reaction (PCR) and droplet digital PCR (ddPCR) provide high analytical sensitivity and rapid turnaround times for detecting predefined alterations in *EGFR*, *KRAS*, and *BRAF* in both tissue and liquid biopsy specimens. However, because these targeted methods require prior knowledge of specific sequence variants, they cannot identify novel structural alterations or broad genomic changes. Furthermore, conventional reverse transcription PCR (RT-PCR) assays face technical limitations in capturing rare or novel fusion partners involving *ALK* or *ROS1*, where RNA-based NGS or FISH is preferred [61]. Meanwhile, FISH remains a highly sensitive method for detecting chromosomal rearrangements, copy number alterations, and gene fusions, particularly involving *ALK* and *ROS1*. Owing to its technical complexity and manual interpretation requirements, FISH is increasingly utilized as a confirmatory method for equivocal results rather than a high-throughput primary screening tool [60]. In contrast, IHC is widely implemented in routine pathology due to its accessibility, cost-effectiveness, and rapid turnaround time, serving as an established primary screening tool for abnormal ALK and ROS1 protein expression. Multiplex IHC (mIHC) further expands spatial tissue characterization by enabling simultaneous evaluation of tumor cells and microenvironmental immune markers, although technical requirements currently limit its routine clinical use [65]. Among these modalities, NGS has become the cornerstone of comprehensive genomic profiling because it enables the simultaneous, high-throughput detection of point mutations, small insertions or deletions, copy number variations, gene fusions, and transcriptomic alterations across multiple genes without prior knowledge of specific variants [60,61]. Crucially, optimal molecular characterization requires distinguishing between DNA-based and RNA-based NGS platforms. While DNA-based NGS reliably identifies single-nucleotide variants (SNVs), small insertions/deletions, and gene amplifications, it exhibits reduced sensitivity for detecting structural gene fusions (e.g., *ALK*, *ROS1*, *RET*, *NTRK1–3*) and splicing alterations due to large, complex intronic regions that are difficult to sequence completely. In contrast, RNA-based NGS sequences mature spliced transcripts, directly demonstrating functional chimeric fusion products and exon-skipping events—such as *MET* exon 14 skipping—regardless of the specific intronic breakpoint location or partner gene involvement. Consequently, integrated DNA/RNA NGS panels represent the clinical gold standard for comprehensive biomarker detection in NSCLC. Despite its broad analytical power, widespread implementation of NGS remains constrained by infrastructure demands, financial costs, nucleic acid input requirements, and bioinformatics complexity. Consequently, contemporary diagnostic algorithms frequently combine rapid screening methods, such as IHC and PCR, with FISH and NGS for confirmatory testing and comprehensive multi-gene profiling, optimizing both diagnostic turnaround time and treatment selection (Table 1) [60].

Importantly, liquid biopsy should be regarded as a complementary rather than a replacement approach to tissue biopsy. While tissue-based molecular profiling remains the standard for the initial identification of actionable genomic alterations, liquid biopsy may provide additional information on temporal changes in tumor biology and the emergence of resistance during treatment. Together, these complementary approaches can support a more comprehensive molecular assessment and facilitate longitudinal monitoring in selected clinical settings.

## 6. Mechanisms of Resistance to Targeted Therapies: A Multi-Tiered Evolutionary Framework

Therapeutic resistance to molecularly targeted therapies in NSCLC is a dynamic evolutionary process driven by continuous selective drug pressure rather than a static molecular event [16,66,67]. Instead of representing isolated mechanisms, resistance arises through interconnected genetic, cellular, and microenvironmental adaptations. This complexity can be conceptualized as a multi-tiered framework comprising target-centric genetic evolution, cellular plasticity, and non-genetic environmental adaptation while integrating both established clinical observations and emerging experimental evidence [68,69].

**Figure 3:**
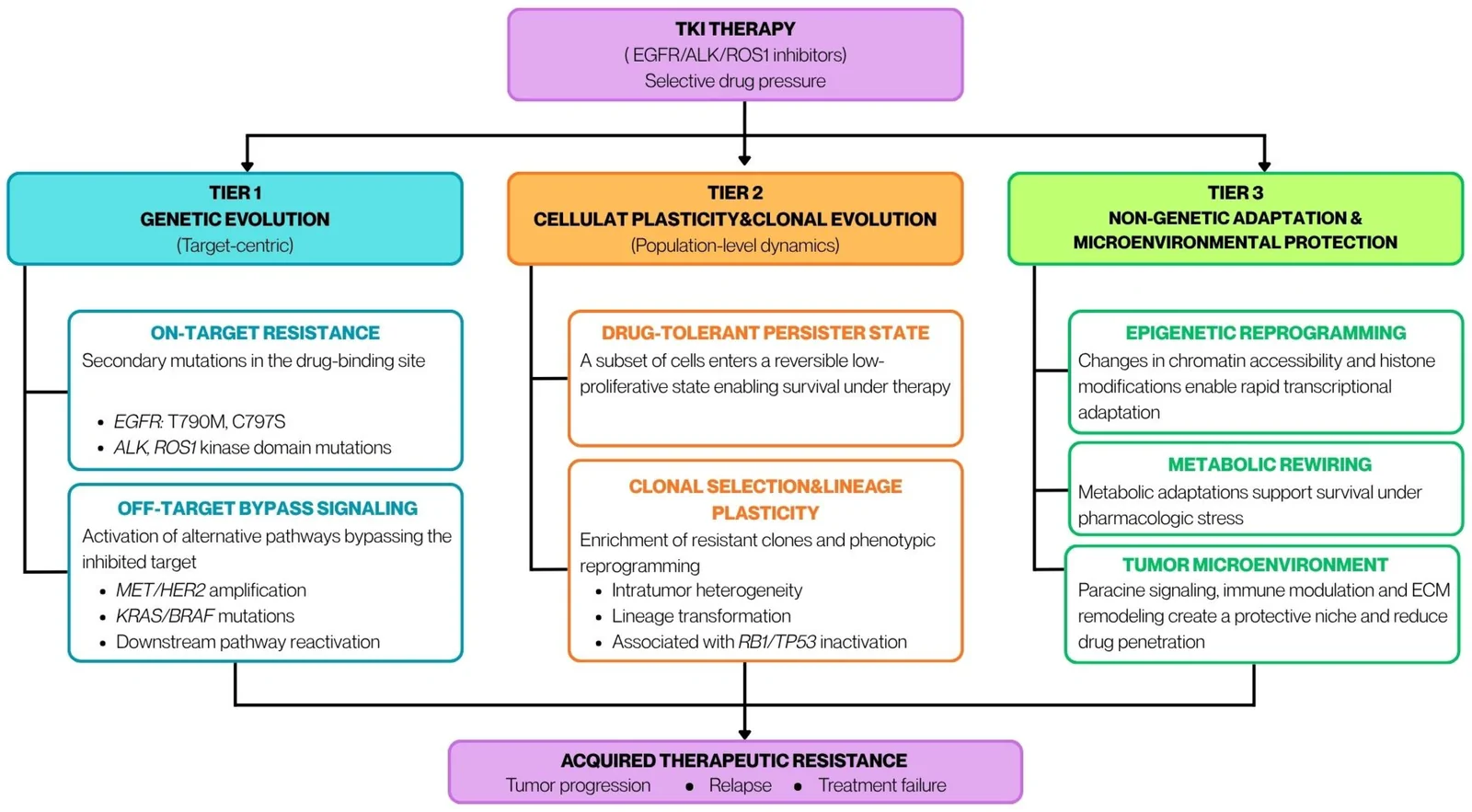
Layered evolutionary mechanisms of resistance to targeted therapies (on the example of *EGFR-*, *ALK*-, or *ROS1*-driven NSCLC). Detailed schematic illustrating the three-tiered progression: Tier 1 (On-target kinase domain mutations and off-target bypass signaling via *MET/HER2/KRAS/BRAF*), Tier 2 (Drug-tolerant persisters, subclonal evolution, and histologic SCLC transformation driven by *RB1/TP53* loss), and Tier 3 (Epigenetic remodeling and TME stromal protective niche).

Clinically, resistance is classified as primary (de novo) or acquired, depending on whether tumors fail to respond initially or progress after an initial objective response [68,69]. However, these categories represent different operational stages of a continuous evolutionary process rather than distinct biological entities (Figure 3).

The first layer (Tier 1) comprises target-centric genetic evolution, encompassing both on-target and off-target resistance mechanisms. On-target resistance results from secondary alterations affecting the drug-binding pocket and reducing inhibitor affinity. In *EGFR*-mutated NSCLC, secondary *EGFR* mutations (e.g., T790M or C797S) restore downstream kinase activity despite continuous therapy [66,68,70,71,72,73]. Similar mechanisms occur in *ALK*- and *ROS1*-rearranged tumors through kinase domain point alterations that directly impair drug binding [74,75,76]. Off-target resistance develops through bypass signaling pathways—such as *MET* or *HER2* amplification—or activating downstream alterations in *KRAS* or *BRAF*, thereby bypassing the inhibited receptor and restoring proliferative and anti-apoptotic signaling cascades [19,70,73].

The second layer (Tier 2) reflects intrinsic intratumoral heterogeneity and cellular plasticity [77,78]. Targeted therapy selectively eliminates sensitive parental clones while allowing pre-existing or emerging resistant subpopulations to expand under therapeutic stress [78,79]. A critical component of this evolutionary dynamic is the drug-tolerant persister state, in which tumor cells survive through reversible epigenetic, transcriptional, and metabolic reprogramming despite the complete absence of genetic resistance mutations [80]. Over time, these persistent cells serve as a reservoir for the acquisition of secondary genetic alterations that lock in permanent resistance. The most profound manifestation of cellular plasticity is histological transformation of *EGFR*-mutated adenocarcinoma into small-cell lung cancer or squamous-cell carcinoma, a process tightly coupled with concurrent *RB1* (retinoblastoma corepressor 1) and *TP53* inactivation, leading to complete loss of sensitivity to target-specific TKIs and unfavorable clinical outcomes [77,81,82,83,84].

The third layer (Tier 3) encompasses non-genetic functional adaptation and microenvironment-mediated resistance mechanisms [85,86,87]. Chromatin remodeling and dynamic metabolic shifts enable tumor cells to rapidly adjust to acute therapeutic stress, while stromal fibroblasts, endothelial cells, and immune components of the tumor microenvironment promote cell survival through paracrine signaling, extracellular matrix remodeling, physical drug shielding, and immunosuppressive barrier formation [85,86,87]. These non-genetic mechanisms contribute to transient or reversible resistance and generate spatial therapeutic response heterogeneity within individual lesions.

From a translational perspective, this multi-tiered framework strongly advocates for biomarker-guided, dynamic therapeutic adaptation over static treatment regimens [88,89]. Multi-target combination strategies—such as combining EGFR-TKIs with selective MET inhibitors, anti-angiogenic agents, chemotherapy, or novel antibody–drug conjugates (ADCs)—have demonstrated high efficacy in suppressing bypass pathway activation and delaying resistance, while next-generation inhibitors targeting specific resistance alterations continue to expand available treatment options [88,90,91,92].

Because resistance evolves continuously across both spatial and temporal dimensions, a single tissue-based molecular profiling may fail to capture the full spectrum of subclonal heterogeneity. In this context, liquid biopsy integrated with high-sensitivity next-generation sequencing serves as an indispensable tool for non-invasive longitudinal monitoring. Real-time quantitative tracking of ctDNA dynamics facilitates the early detection of emerging Tier 1–3 resistance alterations prior to radiological progression, supporting timely therapeutic adaptation and offering deep insights into ongoing intra-patient clonal evolution [55,79,93].

## 7. Future Directions in Precision Oncology for Lung Cancer

The rapid evolution of precision oncology is shifting lung cancer management from single-biomarker testing toward integrated molecular characterization. Although actionable alterations such as *EGFR*, *ALK*, *ROS1*, and *KRAS* have transformed treatment strategies, therapeutic response is increasingly recognized as being influenced by broader genomic, transcriptomic, and microenvironmental contexts. Accordingly, comprehensive molecular profiling using large-panel NGS, together with multidisciplinary Molecular Tumor Boards, may facilitate interpretation of co-occurring alterations that influence therapeutic response and resistance patterns [94].

Growing attention has focused on co-occurring alterations in genes such as *KEAP1*, *STK11*, *SMARCA4* (SWI/SNF related, matrix associated, actin dependent regulator of chromatin, subfamily a, member 4), and *PTEN* (phosphatase and tensin homolog), which may modify sensitivity to immune checkpoint inhibitors (ICIs) by modulating tumor immunogenicity and the tumor microenvironment [95]. Integrating PD-L1 expression, tumor mutational burden (TMB), and co-occurring genomic alterations supports the transition from isolated predictive biomarkers toward composite multi-marker models that better reflect overall tumor biology and refine patient selection for immunotherapy.

Artificial intelligence (AI) is emerging as a cornerstone for integrating the exponentially growing volume of complex multi-omic, clinical, and spatial data in thoracic oncology. Deep learning algorithms, including convolutional neural networks (CNNs), can effectively fuse high-dimensional genomic datasets with quantitative radiomic features derived from computed tomography (CT) and positron emission tomography (PET) imaging, as well as digital pathology tissue metrics [96].

AI-driven predictive models are being actively investigated to decipher composite biomarker signatures, aiming to optimize therapeutic decision-making for both immunotherapy and targeted agents by continuously synthesizing complex molecular landscapes with clinical phenotypes [97].

**Figure 4:**
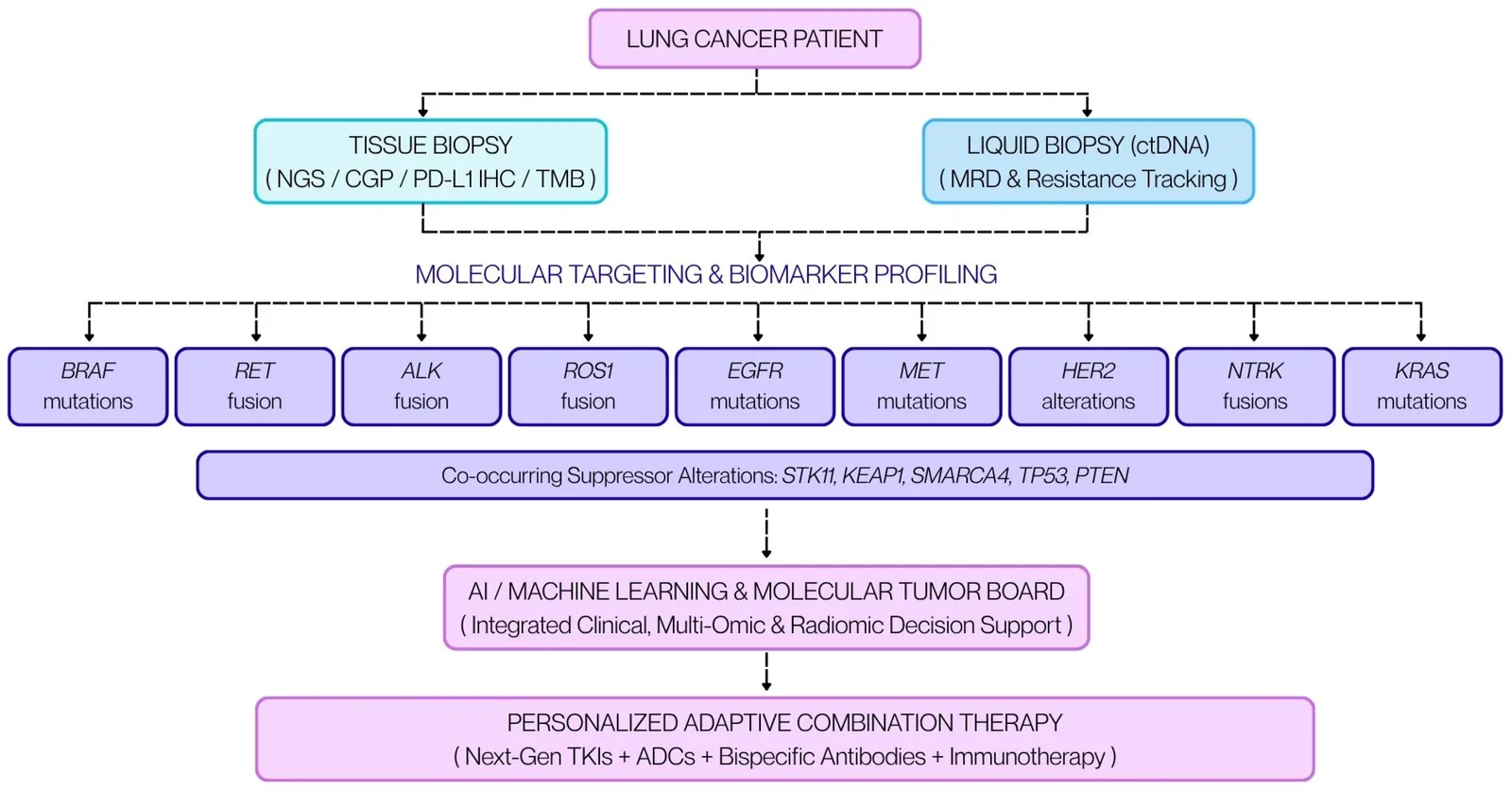
Conceptual framework of future precision oncology in lung cancer: From multi-omic integration to adaptive clinical decision-making. Comprehensive multi-modal platform integrating multi-omic profiling (large-panel NGS targeting actionable drivers such as *EGFR*, *ALK*, *ROS1*, *KRAS*, *MET*, *RET*, and *NTRK*, alongside co-occurring suppressor mutations in *KEAP1*, *STK11*, *SMARCA4*, and *PTEN*), composite biomarker scoring (PD-L1, TMB), digital pathology, and CT/PET radiomics. Convolutional neural networks and deep learning algorithms process these multi-dimensional datasets to inform Molecular Tumor Boards (MTBs). This multi-modal workflow guides targeted therapeutic strategies (next-generation TKIs, antibody–drug conjugates, bispecific antibodies) and directs longitudinal monitoring via serial liquid biopsy (ctDNA) for MRD tracking and early clonal evolution detection prior to radiologic progression [59,95,96,97].

At the therapeutic level, selective small-molecule inhibitors targeting *RET* rearrangements, *MET* exon 14 skipping alterations, *HER2* (ERBB2) mutations or amplifications, and *NTRK* gene fusions continue to expand the scope of molecularly defined treatment populations in non-small cell lung cancer [48,49]. These targeted breakthroughs illustrate the clinical translation of individualized therapy algorithms founded upon broad molecular profiling (Figure 4).

Despite these remarkable advances, acquired resistance remains the principal obstacle limiting durable clinical benefit. Emerging evidence suggests that sustained disease control can be achieved through adaptive treatment paradigms that combine sequential molecular monitoring with rational, biomarker-guided therapeutic combinations—including next-generation kinase inhibitors, antibody–drug conjugates (ADCs), bispecific antibodies, and novel multi-target agents [98,99]. The routine integration of longitudinal molecular monitoring via liquid biopsy into clinical practice will further enable the early detection of resistance-associated subclonal alterations and provide an actionable window for timely therapeutic adaptation before overt clinical or radiological progression occurs [55,59,93,99].

## 8. Conclusions

The management of advanced non-small cell lung cancer has been fundamentally transformed by the integration of comprehensive molecular diagnostics, targeted therapies, and immunotherapy into routine clinical practice. Identification of actionable oncogenic drivers and predictive biomarkers has enabled increasingly personalized treatment selection based on the molecular characteristics of individual tumors.

Nevertheless, durable therapeutic outcomes are influenced not only by isolated genomic alterations, but also by the complex, dynamic processes underlying ongoing tumor evolution—including intratumoral heterogeneity, subclonal selection, epigenetic remodeling, histologic lineage plasticity (such as SCLC transformation), and protective crosstalk within the tumor microenvironment (TME). These layered mechanisms drive acquired treatment resistance and underscore the inherent limitations of single-timepoint, static molecular characterization.

Current evidence supports a paradigm shift from a static, single-biomarker-centered approach toward a dynamic, multi-modal platform for precision oncology that integrates comprehensive baseline multi-omic profiling with longitudinal disease monitoring via serial liquid biopsy (ctDNA). In conjunction with artificial intelligence-driven data integration and Molecular Tumor Boards, such an integrated framework will optimize clinical decision-making, facilitate adaptive combination treatment strategies, and ultimately translate into sustained clinical benefit and improved long-term outcomes for patients with NSCLC.

## Figures and Tables

**Table 1:** Comparison of major diagnostic methods used in non-small cell lung cancer for qualification to molecularly targeted therapies. Adapted from Conde et al. and Saha et al. [60,61].

Method	Target/Alterations Detected	Key Advantages	Major Limitations/Drawbacks
**Real-time PCR/ddPCR**	Specific, predefined point mutations, small insertions/deletions, and target DNA/RNA sequences	High analytical sensitivity, quantitative precision, cost-effective, and rapid turnaround time	Targeted approach requiring prior knowledge of target variants; limited ability to detect novel or partner-agnostic *ALK* and *ROS1* rearrangements
**FISH**	Genomic rearrangements, gene fusions, and gene copy number alterations (*MET*/*HER2* amplifications)	Highly sensitive for chromosomal rearrangements; established reference method for confirming *ALK* and *ROS1* fusions	Technically demanding, labor-intensive, requires specialized fluorescence microscopy, and lacks high-throughput multiplexing
**IHC**	Protein expression, spatial distribution, and biomarker localization within tissue sections	Widely available, low cost, rapid turnaround time; highly effective as an initial screening tool for ALK and ROS1 expression	Conventional single-marker IHC lacks comprehensive genomic profiling capability; mIHC requires costly specialized equipment and complex image analysis
**NGS**	Broad spectrum of genomic and transcriptomic alterations (multi-gene mutations, indels, CNVs, novel gene fusions)	High-throughput, comprehensive multi-gene profiling; enables simultaneous detection of canonical drivers, rare variants, and co-alterations	High upfront cost, requires complex bioinformatic pipelines, dedicated infrastructure, and adequate high-quality tissue input

## Data Availability

Data sharing is not applicable to this article as no new datasets were generated or analysed during the current study.
